# Trimethylamine-N-oxide damages astrocytes and lymphatic endothelial cells in the cerebral lymphatic system

**DOI:** 10.1016/j.ibneur.2025.09.001

**Published:** 2025-09-04

**Authors:** Mei-lan Su, Hai-shui Duan, Qing-lin Wang, Ying Zhang, Xiao-man Shi, Juan Zeng, Wan-ning Tan, Yuan Chang, Song Wang

**Affiliations:** a Department of Psychosomatic and Sleep Medicine, Chongqing University Three Gorges Hospital, Chongqing, China; b Department of Clinical Laboratory, Chongqing University Three Gorges Hospital, Chongqing, China; c Department of Breast surgery, Chongqing University Three Gorges Hospital, Chongqing, China; d Department of Cardiovascular Surgery, Chongqing University Three Gorges Hospital, Chongqing, China

**Keywords:** Trimethylamine-N-oxide, Brain glymphatic system, Meningeal lymphatic vessels, Astrocytes, Lymphatic endothelial cells, NF-κB signaling pathway

## Abstract

**Background:**

Trimethylamine-N-oxide (TMAO), as a gut microbiota dependent metabolite, is involved in the occurrence and progression of many neurodegenerative diseases such as Alzheimer's disease and Parkinson's disease, which are related to the disruption of the cerebral lymphatic system. However, the relationship between TMAO and cerebral lymphatic system remains to be elucidated. This study aimed to investigate the effects of TMAO on the astrocytes of the brain glymphatic system, and endothelial cells of the meningeal lymphatic vessels.

**Methods:**

Astrocytes and lymphatic endothelial cells were treated with different concentrations of TMAO. Alterations in cell proliferation or apoptosis, inflammatory cytokines, the nuclear factor-kappaB (NF-κB) signaling pathway, reactive oxygen species (ROS), and functional proteins such as aquaporin-4 (AQP4), glial fibrillary acidic protein (GFAP), S100β, claudin-5, and Ocln were analyzed. C57BL/6 male mice were treated with TMAO after which alpha-synuclein (SNCA) was injected intracranially. Neuronal damage and expressions of above functional proteins in the ventral midbrain, and levels of SNCA and inflammatory factors in the cerebrospinal fluid (CSF) of mice were assessed.

**Results:**

TMAO activated the NF-κB signaling pathway, increased nucleotide-binding oligomerization domain-like receptor family pyrin domain containing 3 (NLRP3), tumor necrosis factor-alpha, interleukin (IL)-6, IL-1β, and ROS levels in astrocytes and lymphatic endothelial cells and promoted their apoptosis; increased the expression of GFAP and S100β, decreased the expression of AQP4 in astrocytes; decreased the expression of claudin-5 and Ocln in lymphatic endothelial cells. However, NF-κ B signaling pathway inhibitor BAY11–7082 improved the above indicators. Animal studies revealed that TMAO induced intracranial inflammation, affected the expression of functional proteins in the cerebral lymphatic system, and intensified SNCA aggregation in the mouse brain.

**Conclusion:**

TMAO can activate the NF-κB signaling pathway and damage the cellular function of brain glymphatic system and meningeal lymphatic vessels, and promote intracranial inflammation and SNCA deposition in mice, which may be a potential mechanism for TMAO involvement in neurodegenerative diseases.

## Introduction

Dysfunction of the cerebral lymphatic system (including meningeal lymphatic vessels and the cerebral glymphatic system) is involved in the occurrence and development of various neurodegenerative diseases, such as Alzheimer's disease (AD) (Iliff et al., 2012), Parkinson's disease (PD) (Ding et al., 2021), and aging (Lee et al., 2022). Astrocytes and lymphatic endothelial cells are important components of the cerebral lymphatic system; however, there is still insufficient research on the simultaneous in vivo or vitro studies of these two types of cells, including their relationship with gut microbiota and their metabolites.

Meningeal lymphatic vessels are important scavenging channels for metabolic waste in the brain, and some metabolites enter the peripheral lymphatic system directly through meningeal lymphatic vessels after passing through the cerebral glymphatic system (Ahn et al., 2019, Tamura et al., 2020). The perivascular space in the brain is connected to astrocytic foot processes that surround blood vessels; aquaporin-4 (AQP4) in the foot processes is critical for mediating fluid exchange and waste removal. Animals lacking AQP4 in astrocytes showed significant slowing of the flow of cerebrospinal fluid (CSF) around the arteries, resulting in an approximately 70 % reduction in interstitial solute clearance and 55 % reduction in β-amyloid (Iliff et al., 2012). Previous studies suggested that many neurodegenerative diseases are related to dysfunction of the cerebral lymphatic system and brain-gut-microbiota axis (Wong-Guerra et al., 2023, Tsunoda, 2017).

Trimethylamine N-oxide (TMAO) is a gut microbiota-dependent metabolite that is involved in various cardio-cerebrovascular and neurodegenerative diseases (Xu et al., 2022, Li et al., 2018, Mudimela et al., 2022). TMAO activates the nuclear factor-kappaB (NF-κB) signaling pathway and induces the production of vasculitis factors and release of reactive oxygen species (ROS), leading to vascular endothelial cell injury, tube wall destruction, thrombosis, and tube stenosis (Boini et al., 2017, Cheng et al., 2019, Liu et al., 2021). TMAO expression levels in the CSF of patients with AD (Vogt et al., 2018) and in the CSF and blood of patients with PD, were significantly increased (Sankowski et al., 2020) and closely related to the severity and progression of PD motor symptoms (Chen et al., 2020). Therefore, TMAO may be a key gut-derived factor that contributes to the onset of AD and PD. However, to our knowledge, the effect of TMAO on the glymphatic system and meningeal lymphatic vessels have not been reported.

In this study, we hypothesized that TMAO can damage the cerebral lymphatic system by activating the NF-κB signaling pathway, which was preliminarily verified through cell experiments and animal studies.

## Results

### Effect of TMAO on lymphatic endothelial cells

**Fig. 1:**
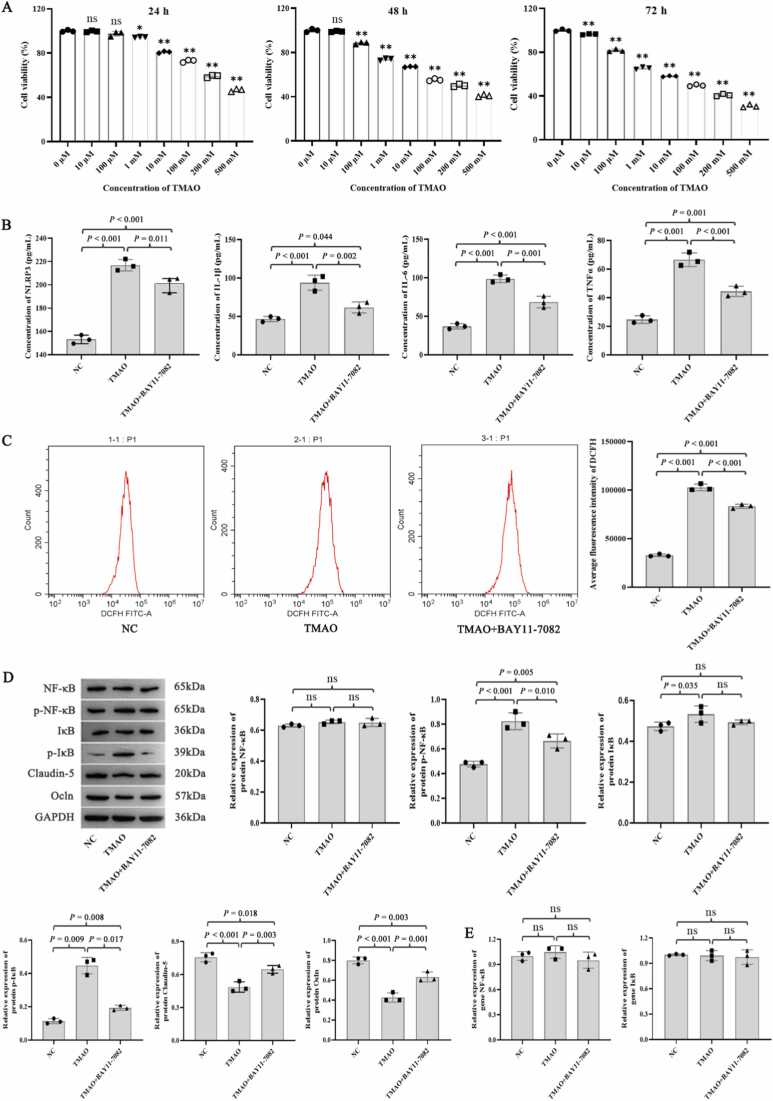
Effect of TMAO on lymphatic endothelial cells. (A) Lymphatic endothelial cells were treated with various concentrations of TMAO and changes in cell viability were detected at 24, 48, and 72 h. The optimal treatment condition for TMAO was 100 µM TMAO for 72 h. (B) Effect of TMAO on the expression of the inflammatory mediators NRLP3, IL-1β, IL-6, and TNF-α in lymphatic endothelial cells. (C) Effect of TMAO on ROS levels in lymphatic endothelial cells. (D) Western blot analysis of IκB, NF-κB, p-IκB, p-NF-κB, claudin-5, and Ocln protein levels. (E) qPCR detection of IκB and NF-κB mRNA levels. N = 3 for each group, analysis of variance followed by least significant difference (LSD) or Dunnett's post-hoc test was used to compare between-group differences. * *P* < 0.05 and ** *P* < 0.01 were compared to control group. 'ns' indicates that there was no significant difference between the groups e.

TMAO at different concentrations (10 µM–500 mM) markedly inhibited the viability of lymphatic endothelial cells at 72 h (*P* < 0.01; Fig. 1A). TMAO at 100 µM had a significant effect on cells and cell viability was high after 72 h; thus, this concentration and exposure time were selected for the subsequent treatment of lymphatic endothelial cells.

In the enzyme-linked immunosorbent assay (ELISA), TMAO treatment significantly increased the levels of nucleotide-binding oligomerization domain-like receptor family pyrin domain containing 3 (NLRP3), interleukin (IL)-1β, IL-6, and tumor necrosis factor (TNF)-α compared with the normal control (NC) group (all *P* < 0.001; Fig. 1B). However, after treatment with the NF-κB signaling pathway inhibitor BAY11–7082, the levels of these inflammatory cytokines decreased significantly compared with those in the TMAO group (all *P* < 0.05). Flow cytometry results showed that the ROS levels in lymphatic endothelial cells were increased significantly by TMAO treatment but decreased by inhibition of the NF-κB signaling pathway (all *P* < 0.001; Fig. 1C).

**Fig. 2:**
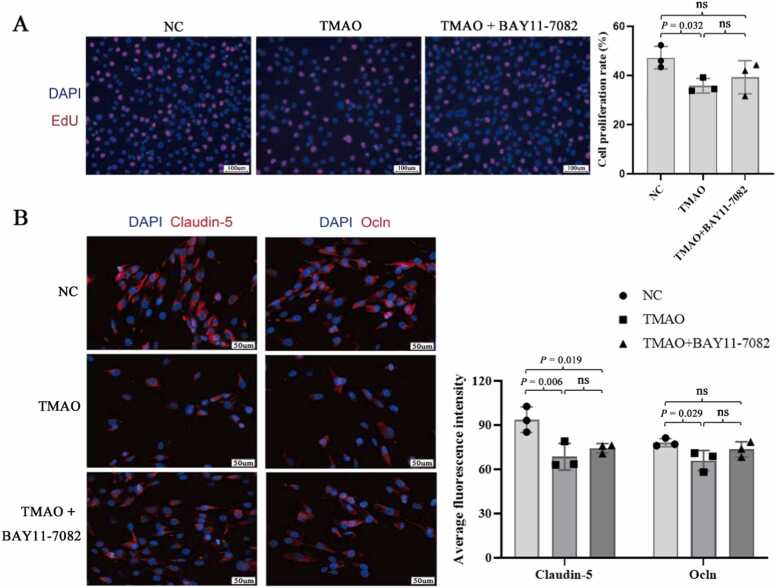
Effect of TMAO on the proliferation and expression of claudin-5 and Ocln in lymphatic endothelial cells. (A) EdU assay detection of cell proliferation. (B) Immunofluorescence detection of changes in the fluorescence intensity of claudin-5 and Ocln. N = 3 for each group, analysis of variance followed by LSD or Dunnett's post-hoc test was used to compare between-group differences. 'ns' indicates that there was no significant difference between the groups.

TMAO significantly increased the protein levels of phosphorylated (p)-IκB and p-NF-κB but decreased with BAY11–7082 treatment (all *P* < 0.05; Fig. 1D). On the contrary, the expression of claudin-5 and Ocln proteins was significantly decreased after TMAO treatment but increased after BAY11–7082 treatment ((all *P* < 0.05; Fig. 1D). Staining experiments revealed that TMAO reduced the proliferation of lymphatic endothelial cells (*P* = 0.032, Fig. 2A) and fluorescence intensity of claudin-5 and Ocln (*P* = 0.006 and 0.029, respectively; Fig. 2B).

### Effect of TMAO on astrocytes

**Fig. 3:**
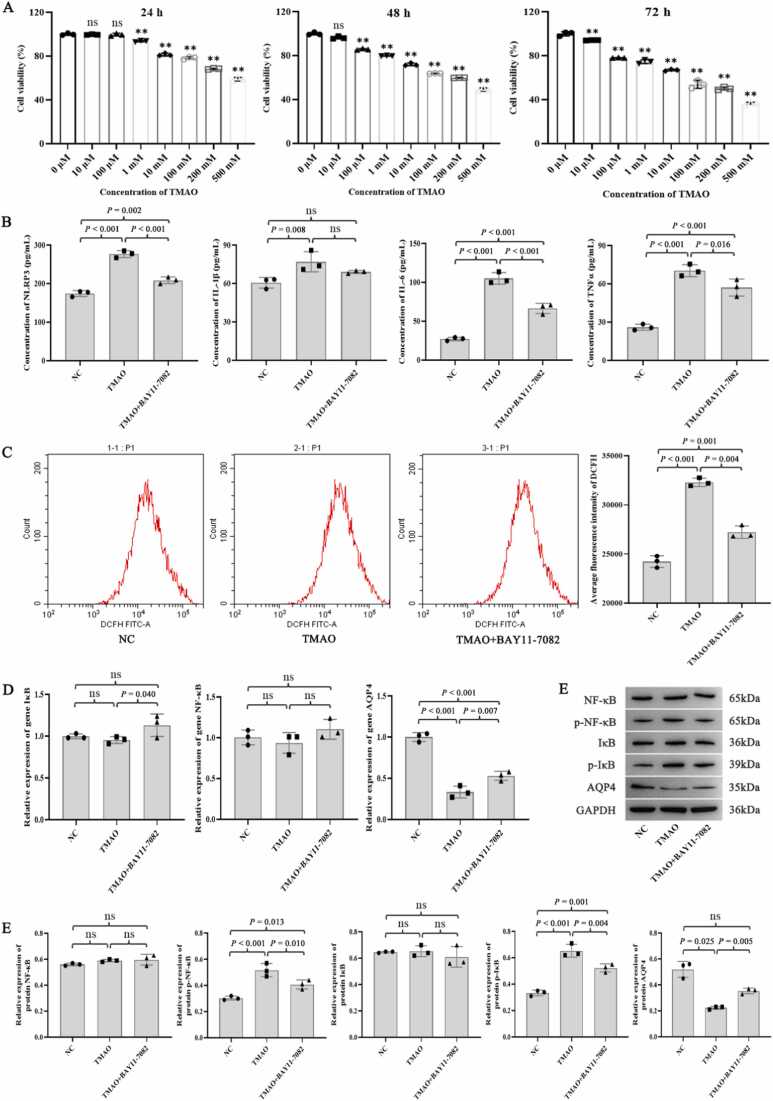
Effect of TMAO on astrocytes. (A) Astrocytes were treated with various concentrations of TMAO and changes in cell viability were detected at 24, 48, and 72 h. The optimal treatment condition for TMAO was 100 µM TMAO for 48 h. (B) Effect of TMAO on the expression of the inflammatory mediators NRLP3, IL-1β, IL-6, and TNF-α in astrocytes. (C) Effect of TMAO on ROS levels in astrocytes. (D) qPCR detection of IκB, NF-κB, and AQP4 mRNA levels. (E) Western blotting analysis of IκB, NF-κB, AQP4, p-IκB, and p-NF-κB protein levels. N = 3 for each group, analysis of variance followed by LSD or Dunnett's post-hoc test was used to compare between-group differences. * *P* < 0.05 and ** *P* < 0.01 were compared to control group. 'ns' indicates that there was no significant difference between the groups.

Different concentrations (10 µM–500 mM) of TMAO markedly inhibited astrocyte viability at 48 and 72 h (*P* < 0.01; Fig. 3A). TMAO at 100 µM had a significant effect on cells after 48 h of treatment, and the cell viability was relatively high; thus, this concentration and exposure time were selected for the subsequent treatment of astrocytes.

After TMAO treatment, the astrocytes exhibited significantly increased levels of NLRP3, IL-1β, IL-6, and TNF-α (all *P* < 0.01; Fig. 3B) compared with those in the NC group, but these levels decreased significantly after inhibiting the NF-κB signaling pathway using BAY11–7082 (all *P* < 0.05, except for IL-1β; Fig. 3B). Compared with the NC group, TMAO significantly increased the fluorescence intensity of 2`,7`-dichlorodihydrofluorescein (*P* < 0.001), indicating increased intracellular ROS levels and an enhanced oxidative stress response. In contrast, the NF-κB signaling pathway inhibitor BAY11–7082 significantly reduced intracellular ROS levels (*P* = 0.004; Fig. 3C). In addition, TMAO significantly increased the protein levels of p-NF-κB and p-IκB (Fig. 3E) and significantly decreased the mRNA and protein levels of AQP4, which was reversed in the presence of BAY11–7082 (all *P* < 0.05; Fig. 3D–3E).

**Fig. 4:**
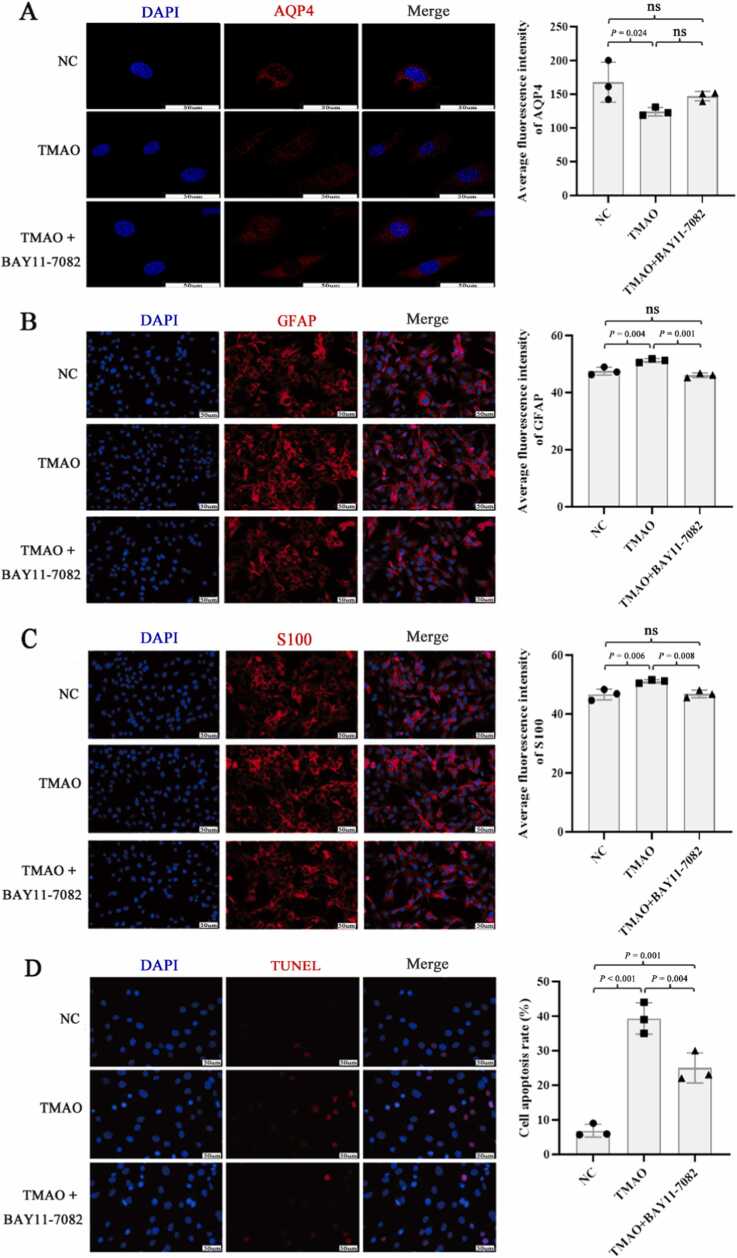
Effect of TMAO on the proliferation and expression of AQP4, GFAP, and S100 in astrocytes. (A–C) Immunofluorescence detection of changes in the fluorescence intensity of AQP4, GFAP, and S100. (D) TUNEL assay detection of cell apoptosis. N = 3 for each group, analysis of variance followed by LSD or Dunnett's post-hoc test was used to compare between-group differences. 'ns' indicates that there was no significant difference between the groups.

Immunofluorescence results showed that TMAO reduced the expression of AQP4 (*P* = 0.024) and significantly increased the expression of GFAP and S100 in astrocytes compared with the control group (*P* = 0.004 and 0.006, respectively; Fig. 4A–4 C). TMAO also significantly promoted astrocyte apoptosis (*P* < 0.001; Fig. 4D). Treatment with BAY11–7082 decreased the fluorescence intensity of GFAP and S100, and inhibited astrocyte apoptosis (all *P* < 0.01; Fig. 4B–4D).

### TMAO increases the inflammatory level in the CSF

Overaggregation of alpha-synuclein (SNCA) and its subsequent pathological cascades are critical in the onset and progression of PD. Therefore, this study investigated the effect of TMAO on the clearance of SNCA in the brain, as well as the impact of SNCA aggregation on neurons in the ventral midbrain of the C57BL/6 mice.

**Fig. 5:**
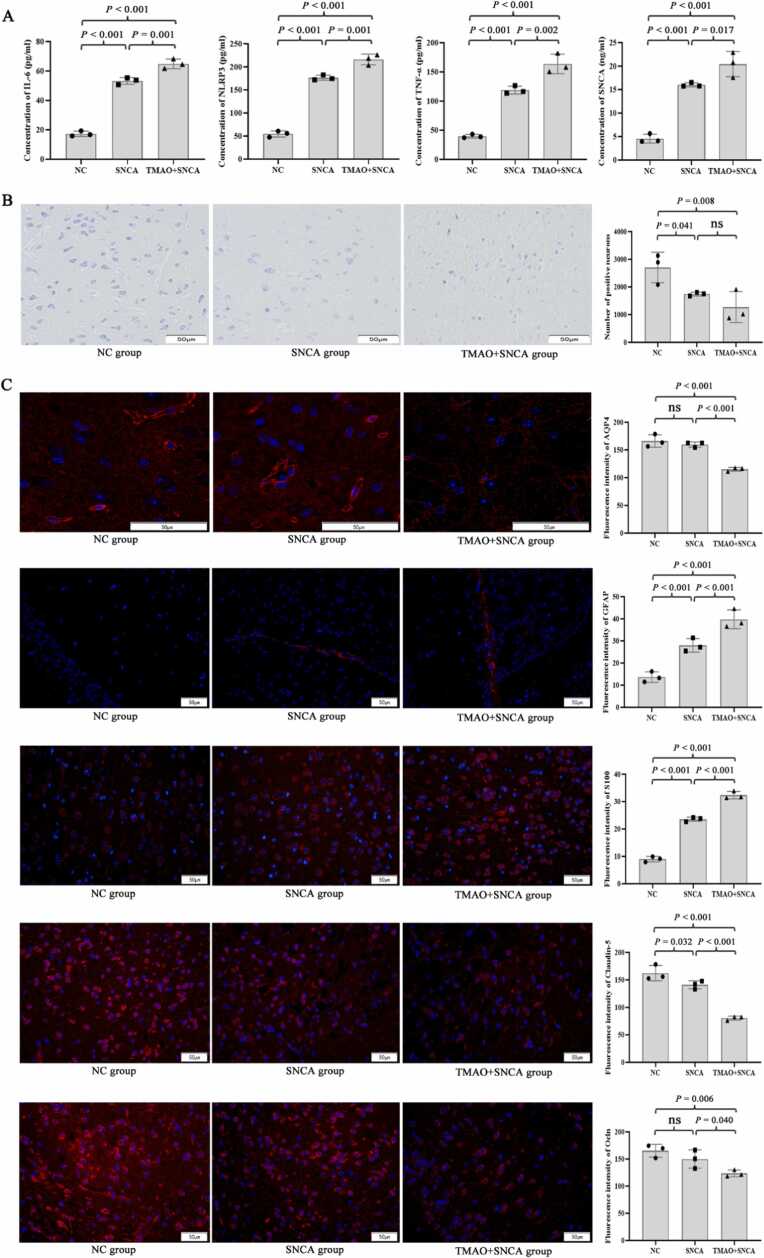
Effects of TMAO on intracranial inflammation and functional proteins in animals. Levels of IL-6, NLRP3, TNF-α, and SNCA in the cerebrospinal fluid detected by ELISA. (B) The injury of ventral midbrain neurons detected by Nissl staining. (C) Immunofluorescence was used to detect the effects of TMAO on AQP4, GFAP, S100, claudin-5, and Ocln in the ventral midbrain of mice. Three animals were randomly selected from each group (n = 10) for sample testing, analysis of variance followed by LSD or Dunnett's post-hoc test was used to compare between-group differences. 'ns' indicates that there was no significant difference between the groups.

After 4 weeks of treatment with TMAO and 30 min after injection of recombinant SNCA, ELISA results for the C57BL/6 mice indicated that expressions of IL-6, NLRP3, TNF-α, and SNCA in the CSF of the TMAO + SNCA treatment group were significantly increased compared to the control group and the SNCA treatment alone (all *P* < 0.05). In addition, the above indicators in the CSF were significantly increased after SNCA treatment compared to the control group (all *P* < 0.001, Fig. 5A).

Nissl staining showed that SNCA or TMAO + SNCA treatment reduced the number of positive cells in midbrain neurons of mice compared to the control group (*P* = 0.041 and 0.008, respectively, Fig. 5B). However, no similar difference was observed between the SNCA group and the TMAO + SNCA group (*P* > 0.05). These results confirm that SNCA induces intracranial inflammation and neuronal damage in mice; and suggest that TMAO induces intracranial inflammation, exacerbates intracranial SNCA aggregation, but may cause less damage to midbrain neurons.

### TMAO damages the brain lymphatic system of mice

Fluorescence staining results showed that, compared to the control group and the group treated with SNCA alone, the fluorescence intensity of AQP4 in the ventral midbrain of mice significantly reduced after treatment with TMAO + SNCA (all *P* < 0.001, Fig. 5C). Moreover, compared to the control group, the fluorescence intensity of GFAP and S100 in the ventral midbrain of the C57BL/6 mice significantly increased after treatment with SNCA or TMAO + SNCA (all *P* < 0.001), whereas the fluorescence intensity of claudin-5 decreased (*P* = 0.032). Simultaneously, the expressions of GFAP and S100 were significantly increased but claudin-5 decreased in the TMAO + SNCA treatment group compared to the SNCA treatment group (all *P* < 0.001, Fig. 5C).

## Discussion

The cerebral lymphatic system plays an important role in the clearance of metabolic waste in the brain. TMAO is involved in the progression of neurodegenerative diseases such as AD (Vogt et al., 2018, Qu et al., 2023, Huguenard et al., 2023) and PD (Sankowski et al., 2020, Chen et al., 2020, Voigt et al., 2022, Zhou et al., 2023), but its effects on the cerebral lymphatic system have been poorly reported. This study provides important clues for clarifying the relationship between TMAO and the destruction of the cerebral lymphatic system.

TMAO, as a small molecule substance, can freely pass through the blood-brain barrier (BBB) (Caradonna et al., 2024). The activation of inflammatory signaling pathways such as NF-κB and NLRP3, and the release of ROS may be the main mechanism of TMAO's involvement in cardiovascular and cerebrovascular diseases (Liu et al., 2022, Praveenraj et al., 2022). The present study showed that high TMAO levels stimulated the expression of inflammatory mediators (including NLRP3, IL-1β, IL-6, TNF-α, p-NF-κB and P-IκB) in astrocytes and lymphatic endothelial cells, induced oxidative stress, promoted astrocyte apoptosis, and reduced lymphatic endothelial cell proliferation. However, these damages were alleviated by the NF-κB signaling pathway inhibitor. TMAO has been widely reported to be involved in the progression of neurodegenerative diseases by activating inflammatory pathways (Caradonna et al., 2024, Praveenraj et al., 2022). Our results indicate that TMAO can not only induce intracranial inflammatory responses but also affect the expression of functional proteins in the cerebral lymphatic system in mice.

Functional regulation of the brain glymphatic system is mainly dependent on AQP-4 in the endfeet of astrocytes in the brain (Iliff et al., 2012). Dysfunction of the glymphatic system, probably related to disrupted AQP4 expression, has been observed in animal models of AD and stroke (Rasmussen et al., 2018). It is reported (Buawangpong et al., 2021, Brunt et al., 2021) that TMAO activates astrocytes and causes inflammatory responses involved in cognitive decline and AD. Our intervention study in vitro showed that TMAO significantly reduced AQP4 expression, which was increased again by the addition of NF-κB inhibitors. GFAP and S100 are important biomarkers of astrocytes, often showing elevated expression in many neurodegenerative diseases such as PD, AD, multiple system atrophy, and dementia with Lewy bodies (Schulz et al., 2021). Our results revealed that TMAO increased the expression of GFAP and S100 in astrocytes, as well as the expression of GFAP and S100 in the midbrain of mice, and also promoted intracranial SNCA deposition. These results suggest that high TMAO levels may activate the NF-κB signaling pathway and damage the physiological function of the brain glymphatic system, thereby affecting the clearance efficiency of toxic proteins such as SNCA in the brain (Buccellato et al., 2022).

Previous studies (Louveau et al., 2015, Aspelund et al., 2015) found that the meningeal lymphatic structure expressed all the molecular characteristics of peripheral lymphatic endothelial cells. The junctions between the lymphatic endothelial cells are mainly composed of some tight junction-related proteins and vascular endothelial cadherins (Baluk et al., 2007), including claudin-5 and Ocln (Baluk and McDonald, 2022). It is reported that the functional network of meningeal lymphatic vessels exists in every corner of the brain, including the midbrain (Chang et al., 2023). In both in vivo and in vitro studies, claudin-5 and Ocln expressions decreased significantly under the action of high TMAO levels compared to the control group. It’s reported that high TMAO concentrations can also cause tight connections in the BBB to loosen (Hernandez et al., 2022), thereby damaging its integrity and affecting transport through the BBB to clear metabolic wastes, such as SNCA and amyloid β, in the brain (Verheggen et al., 2018). Evidently, our research results revealed that TMAO can affect intracranial SNCA clearance by disrupting cerebral lymphatic system, rather than just the BBB.

However, there are also some limitations in this study that warrant elaboration. Firstly, a separate control study for the NF-κB inhibitor BAY11–7082 was not established in the cell experiments, although the effect of BAY11–7082 on normal cells might be minimal (Mori et al., 2002). Secondly, the effects of multiple concentrations of TMAO on the brain lymphatic system and behavioral changes in mice were not observed in animal experiments. These deficiencies will be further improved in future research.

## Conclusion

These results confirm the destructive effects of TMAO on astrocytes and lymphatic endothelial cells through in vivo and in vitro studies. TMAO may impair the function of the cerebral lymphatic system by promoting inflammatory response and release of ROS, which may be a potential mechanism for TMAO involvement in the progression of neurodegenerative diseases such as PD. Clinical studies on the effects of TMAO on the cerebral lymphatic system are a valuable research direction in the future.

## Materials and methods

### Cell culture and passage

Mouse cerebral cortical astrocytes (CP-M157) and lymphatic endothelial cells (CP-M023) were purchased from ProCell (China). The two cell lines were incubated in Dulbecco's modified Eagle’s medium (DMEM)/F12 complete culture medium containing 10 % fetal bovine serum (AC03L055; Life-iLab, China), 1 % penicillin-streptomycin, and 1 % dedicated growth factors at 37 °C with 95 % O_2_ and 5 % CO_2_. When the cells reached a fusion rate of 80–90 %, they were passaged at a 1:2 ratio, and the third generation of cells was used for subsequent experiments. A Mycoplasma Staining Assay Kit (C0296; Beyotime) was used to confirm that the cells were not contaminated with mycoplasma.

### Selection of TMAO concentration

TMAO (S25980; Yuan Ye, China) was prepared at eight different concentrations (of 0, 10 µM, 100 µM, 1 mM, 10 mM, 100 mM, 200 mM, and 500 mM) in phosphate buffered saline (PBS). Astrocytes or lymphatic endothelial cells were seeded into a 96-well plate at 100 μL/well (1 ×10^4^ cells) and treated with 10 μL of TMAO at different concentrations. Subsequently, 10 μL of Cell Counting Kit-8 reagent (CCK-8, CA1201; Solarbio, China) was added at 24 h, 48 h, and 72 h and incubated for 2 h. The absorbance at 450 nm was measured using an enzymatic calibrator to determine the concentration and treatment time of TMAO by measuring the cell viability.

### Cell proliferation assays by EdU

A 5-Ethynyl-20-Deoxyuridine (EdU) test kit (C0071S; Beyotime) was used to detect the proliferative capacity of lymphatic endothelial cells. The concentration of the EdU working solution was 20 μm/mL, and 1 mL of the solution was added to the cells with different treatments. After incubation for 2 h, EdU labeling of the cells was performed. The culture medium was then discarded. Subsequently, 1 mL of 4 % paraformaldehyde was added and the cells were fixed for 15 min at room temperature. After incubation with 0.3 % TritonX-100 (ST795; Beyotime) and DAPI staining solution (C1005; Beyotime), EdU-positive cells were observed under a fluorescence microscope (MF53, Mingmei Optoelectronic Technology Co., Ltd, China).

### Cell apoptosis by TUNEL assay

Astrocyte apoptosis was detected using a TUNEL assay kit. After the treated cells were digested with pancreatic enzymes, the suspension was adjusted to a density of 1 × 10^6^ /mL in DMEM/F12 medium. Three cell slides were placed in a dish, and cell suspension was added dropwise and incubated at 37 °C in an incubator with 95 % O_2_ and 5 % CO_2_ for 3 h. PBS containing 0.3 % Triton X-100 was added dropwise for 5 min and incubated at 37 °C for 1 h with a TUNEL assay solution (TdT enzyme: fluorescent labeling solution = 1:9). The slides were then washed three times with PBS, stained with DAPI for 5 min, and sealed with an anti-fluorescence quenching agent (P0126; Beyotime). TUNEL-positive cells were observed using a fluorescence microscope (MF53, Mingmei Optoelectronic Technology Co., Ltd, China).

### Cell grouping and intervention

The lymphatic endothelial cells and astrocytes were randomly divided into the following three groups: (1) normal control (NC) group, cultured with DMEM/F12 complete media; (2) TMAO group, cultured with DMEM/F12 complete media containing 100 µM TMAO; (3) TMAO + BAY11–7082 group, cultured with DMEM/F12 complete media containing 100 µM TAMO and 2.5 µM BAY11–7082. Based on previous experiments, the astrocyte group was cultured at 37 °C, 95 % O_2_, and 5 % CO_2_ for 48 h, and the lymphatic endothelial cell group was cultured under the same conditions for 72 h.

### ROS by flow cytometry

Diacetyldichlorofluorescein (DCFH-DA) (S0033S; Beyotime) was diluted 1:1000 using DMEM/F-12 base media. After culturing the cells by grouping, the medium was discarded and an appropriate amount of DCFH-DA was added to each well (a volume sufficient to adequately cover the cells). The cells were then incubated for 20 min at 37 °C with 95 % O_2_ and 5 % CO_2_. The cells were washed three times with DMEM/F-12 base medium to remove the DCFH-DA that had not entered the cells. The fluorescence intensity of DCF was determined using a flow cytometer (CytoFLEX, Beckman Coulter, USA), allowing the identification of intracellular ROS levels.

### Quantitative polymerase chain reaction (qPCR)

Total RNA was extracted from the cells using an RNA extraction kit (RC112, Vazyme, China). After the RNA concentration was determined, the reaction system was configured according to the reverse transcription kit (TSK302S; Tsingke Biotech, China), and the reaction was performed at 25 °C for 10 min, 55 °C for 30 min, and 85 °C for 5 min to synthesize cDNA. The qPCR system was configured using a SYBR Green kit (TSK202; Tsingke Biotech) in a real-time fluorescence quantitative PCR instrument (IQ5; Bio-Rad, USA). The qPCR assay was performed under the following thermal cycling conditions: 40 cycles of 95 °C for 30 s, 95 °C for 5 s, and 60 °C for 30 s. The primers used are listed in Table 1.

**Table 1:** Primer sequences for qPCR.

Genes	Sequences (5’-3’)
NF-κB	Forward: ACACCTCTGCATATAGCGGC Reverse: GCAGAGTTGTAGCCTCGTGT
IκB	Forward: ACTTCTCCTGAAAGCCGGTG Reverse: AGGAAGAGGTTTGGATGCCG
GAPDH	Forward: GGAGAGTGTTTCCTCGTCCC Reverse: TTTGCCGTGAGTGGAGTCAT

### Animal grouping and intervention

The C57BL/6 male mice (SPF grade), 6-weeks old and weighing 12–18 g, were supplied by Ensiweier Biotechnology Co., Ltd. (Chongqing, China) and housed at 25°C, with humidity between 35 % and 60 %, under a 12-h light-dark cycle. Food and water were provided ad libitum to all animals. The animals were reared normally and acclimatised for one week before pharmacological intervention. According to the intervention methods, mice were randomly categorized into three groups: NC group, SNCA group, and TMAO + SNCA group, with at least 10 mice in each group. The animal experiment was approved by the Ethics Committee of Chongqing University Three Gorges Hospital and conducted according to the principles of the Declaration of Helsinki.

Mice requiring TMAO intervention were given intraperitoneal injections of 200 mM TMAO (S25980; Yuan Ye, China) once daily at a dose of 1.5 g /kg (1 mL /10 g) mouse weight for 4 weeks, as previously described (Ke et al., 2018). Mice not requiring TMAO were treated with an equal volume of normal saline. After TMAO administration, mice were anesthetized with ketamine at a dose of 100 mg/kg by intraperitoneal injection, then stereotactically injected with 2.5 μL (concentration 3 μg/mL) recombinant human SNCA protein (PRS-pro-393, Amyjet Scientific Technology Co., Ltd, Hubei, China) intracranially at a rate of 0.25 μL/min. The injection was maintained for 2 min to avoid fluid leakage. All experiments were repeated at least once.

### Collection of CSF and tissue samples

Three randomly selected mice in each group were euthanized with 0.3 % pentobarbital sodium (P3761, BSZH Scientific LLC, Beijing, China) at a dose of 200 mg/kg, and CSF samples were collected. Then their thoracic cavity was opened to expose the heart. A 1-mL syringe was inserted into the left ventricle. The right atrium and liver were cut, perfused with normal saline to drain the blood until the liquid from the right atrium became colorless and transparent, and then perfused with a 4 % polyformaldehyde solution. The ventral midbrain was removed and placed in a 4 % polyformaldehyde solution for fixation. The fixed tissue was washed with PBS, dehydrated using a series of gradient ethanol, embedded in paraffin, and prepared into 4-μm slices.

### Enzyme-linked immunosorbent assay (ELISA)

Cytokine levels were determined using ELISA. The supernatant was collected after cell treatment. The levels of NLRP3, IL-1β, IL-6, and TNF-α in the cell samples were analyzed according to the manufacturer's instructions (Mlbio, China). Similarly, the levels of NLRP3, IL-1β, IL-6, TNF-α, and SNCA in the CSF samples were determined using ELISA, and were analyzed according to the manufacturer's instructions (Ruixin Biotech, China). Each plate contained a recombinant mouse cytokine standard curve and known positive and negative controls. All measurements were repeated at least three times and averaged.

### Immunofluorescence

The main steps of immunofluorescence were as follows: cell or tissue preparation, fixation, permeabilization, sealing, incubation of primary antibody, incubation of secondary antibody, cell nucleus staining, and sealing, etc. The primary antibodies used include S100 (1: 100, bs-1248R, Bioss, China), glial fibrillary acidic protein (GFAP, 1: 100, R380620, Zenbio, China), AQP4 (1: 200, A11210, ABclonal, China), claudin-5 (1: 200, A10207, ABclonal, China), and Ocln (1: 200, YN2865, Immunoway, China), and the secondary antibody was goat anti-rabbit IgG (1: 500, 550076, Zenbio, China). Finally, DAPI was used to stain for 5 min in the dark and the slides were sealed with an anti-fluorescence quenching agent and observed under a fluorescence microscope (MF53, Mingmei Optoelectronic Technology Co., Ltd, China), and quantification was performed using ImageJ software.

### Nissl staining

The Nissl staining solution (SL7810, Coolaber, China) was used for the Nissl staining assay. The ventral midbrain sections were placed into a Cresyl Violet Stain and heated until steaming (avoiding boiling). After rinsing with distilled water, the sections were immediately immersed in Nissl Differentiation solution for 2 min and observed under a microscope until the background faded. The slides were soaked and dehydrated in anhydrous ethanol, rendered transparent using xylene, and sealed with neutral resin. Finally, Nissl bodies were observed under a microscope.

### Western blotting

Radioimmunoprecipitation assay lysis buffer was premixed with 1 mM phenylmethylsulfonyl fluoride and a protease inhibitor cocktail to lyse the cells and extract total proteins. Protein concentrations were determined using a BCA Protein Assay Kit (PC0020; Solarbio, China). For each western blot analysis, the same amount of total protein (60 µg) was isolated using 8–12 % sodium dodecyl-sulfate polyacrylamide gel electrophoresis and electrically transferred to a polyvinylidene fluoride membrane, which was blocked with 5 % skimmed milk for 1 h at room temperature. The membrane was incubated overnight with the following primary antibodies: NF-κB (1:500, 10745–1-AP; Proteintech, USA), phospho-NF-κB (1:1000, 3033 T; Cell Signaling Technology, USA), IκB (1:1000, A11397; ABclonal, China), phospho-IκB (1:1000, AP0731; ABclonal), claudin-5 (1:2000, A10207; ABclonal), occludin (Ocln) (1:500, YN2865; Immunoway, China), AQP4 (1:500, A2887; ABclonal), and GAPDH (1:10000, A19056; ABclonal). The secondary antibody, goat anti-rabbit IgG (1:2000, AS014; ABclonal), was incubated for 1 h at room temperature. The bands were detected using an ECL kit (34580; Thermo, USA) and a Universal Hood II protein imaging system (Bio-Rad, USA). The gray values of the bands were analyzed using the ImageJ software (ver1.48, National Institutes of Health, USA), and the relative protein expression was calculated using GAPDH normalization.

## Statistical analysis

Statistical analyses of all clinical data and scale scores were performed using the Statistical Package for the Social Sciences (SPSS 23.0). Measurement data are expressed as the mean ± standard deviation. The two-sample *t*-test or analysis of variance followed by least significant difference (LSD) or Dunnett's post-hoc test was used to compare continuous variables with group differences where data were normally distributed, whereas the Mann–Whitney *U* test or Kruskal–Wallis H test was used to compare the distribution of variables between the groups. Statistical significance was set at *P* < 0.05.

## CRediT authorship contribution statement

**Duan Haishui:** Supervision, Resources, Investigation, Conceptualization. **Su Meilan:** Writing – original draft, Project administration, Methodology, Funding acquisition, Data curation. **Ying Zhang:** Validation, Data curation. **Wang Qinglin:** Validation, Resources, Methodology, Data curation. **Juan Zeng:** Investigation, Data curation. **Shi Xiaoman:** Resources, Investigation. **Yuan Chang:** Resources, Investigation. **Tan Wanning:** Resources, Data curation. **Song Wang:** Writing – review & editing, Supervision, Methodology, Funding acquisition, Conceptualization.

## Conflicts of Interest

All authors declare no potential conflicts of interest with respect to the research, authorship, and publication of this article. The animal experiment was approved by the Ethics Committee of Chongqing University Three Gorges Hospital (Review Number: SXYYWD2022–056) and conducted according to the principles of the Declaration of Helsinki.
